# Comparative expression profiling of *E. coli *and *S. aureus *inoculated primary mammary gland cells sampled from cows with different genetic predispositions for somatic cell score

**DOI:** 10.1186/1297-9686-43-24

**Published:** 2011-06-24

**Authors:** Bodo Brand, Anja Hartmann, Dirk Repsilber, Bettina Griesbeck-Zilch, Olga Wellnitz, Christa Kühn, Siriluck Ponsuksili, Heinrich HD Meyer, Manfred Schwerin

**Affiliations:** 1 Research Group of Functional Genomics, Leibniz Institute of Farm Animal Biology, 18196 Dummerstorf, Germany; 2 Research Unit of Genetics and Biometry, Leibniz Institute of Farm Animal Biology, 18196 Dummerstorf, Germany; 3 Research Unit of Molecular Biology, Leibniz Institute of Farm Animal Biology, 18196 Dummerstorf, Germany; 4 Institute of Physiology, Technical University Munich, 85350 Freising, Germany; 5 Veterinary Physiology, Vetsuisse Faculty, University of Bern, 1725 Posieux, Switzerland; 6 Institute of Farm Animal Science and Technology, University of Rostock, 18059 Rostock, Germany

## Abstract

**Background:**

During the past ten years many quantitative trait loci (QTL) affecting mastitis incidence and mastitis related traits like somatic cell score (SCS) were identified in cattle. However, little is known about the molecular architecture of QTL affecting mastitis susceptibility and the underlying physiological mechanisms and genes causing mastitis susceptibility. Here, a genome-wide expression analysis was conducted to analyze molecular mechanisms of mastitis susceptibility that are affected by a specific QTL for SCS on *Bos taurus *autosome 18 (BTA18). Thereby, some first insights were sought into the genetically determined mechanisms of mammary gland epithelial cells influencing the course of infection.

**Methods:**

Primary bovine mammary gland epithelial cells (pbMEC) were sampled from the udder parenchyma of cows selected for high and low mastitis susceptibility by applying a marker-assisted selection strategy considering QTL and molecular marker information of a confirmed QTL for SCS in the telomeric region of BTA18. The cells were cultured and subsequently inoculated with heat-inactivated mastitis pathogens *Escherichia coli *and *Staphylococcus aureus*, respectively. After 1, 6 and 24 h, the cells were harvested and analyzed using the microarray expression chip technology to identify differences in mRNA expression profiles attributed to genetic predisposition, inoculation and cell culture.

**Results:**

Comparative analysis of co-expression profiles clearly showed a faster and stronger response after pathogen challenge in pbMEC from less susceptible animals that inherited the favorable QTL allele 'Q' than in pbMEC from more susceptible animals that inherited the unfavorable QTL allele 'q'. Furthermore, the results highlighted *RELB *as a functional and positional candidate gene and related non-canonical Nf-kappaB signaling as a functional mechanism affected by the QTL. However, in both groups, inoculation resulted in up-regulation of genes associated with the Ingenuity pathways 'dendritic cell maturation' and 'acute phase response signaling', whereas cell culture affected biological processes involved in 'cellular development'.

**Conclusions:**

The results indicate that the complex expression profiling of pathogen challenged pbMEC sampled from cows inheriting alternative QTL alleles is suitable to study genetically determined molecular mechanisms of mastitis susceptibility in mammary epithelial cells *in vitro *and to highlight the most likely functional pathways and candidate genes underlying the QTL effect.

## Background

Mastitis or the inflammation of the mammary gland has the highest economical impact of all productive diseases in dairy cattle [1]. In addition to the economical losses in milk production, the negative effects on animal welfare as well as food-born pathogens that can cause potential damage to human health are the main reasons for intensive research on this topic during the last decades [2]. So far, many studies have identified genomic regions harboring quantitative trait loci (QTL) affecting clinical mastitis or mastitis-related traits [3,4]. The number of studies investigating molecular mechanisms of immune response to different mastitis pathogens *in vivo *and *in vitro *in cattle is also increasing [5-10]. However, the link between QTL, causal mutations affecting the phenotypic variation in mastitis susceptibility and how these mutations alter or affect molecular mechanisms is still lacking for most QTL. So far, only a few studies have investigated molecular mechanisms affected by a QTL for udder health or related traits [11].

In a first study [12], we demonstrated the suitability of an *in vitro *test system to investigate the transcriptome of primary mammary epithelial cells. In the present study, we conducted a genome-wide expression analysis to analyze the molecular mechanisms of mastitis susceptibility in cattle that are affected by a specific QTL on *Bos taurus *autosome 18 (BTA18). Several reports have shown that BTA18 harbors QTL affecting clinical mastitis or mastitis-related traits like the somatic cell score (SCS) in the German Holstein [13-17] and other cattle populations [18-21]. SCS, a phenotypic measure of the number of somatic cells in milk, is often used as a surrogate trait for udder health and has a strong genetic correlation to mastitis in the German Holstein population (r_g _= 0.84; [22]). One of the best confirmed QTL affecting SCS in the German Holstein population is located at the telomeric end of BTA18 (hereinafter referred to as SCS-BTA18-QTL) [13,16,17]. Within this region, QTL affecting udder conformation traits like fore udder attachment and udder depth have also been reported [23,24], traits that are known to have a substantial impact on udder health [25]. Thus, the specific functional background underlying the SCS-BTA18-QTL could not be unambiguously inferred, because aside from mechanisms of immune defense, udder conformation might also contribute to the genetic variability of mastitis susceptibility. Additionally, the chromosomal region enclosing the QTL confidence interval is characterized by a high gene density [26]. Thus, the aim of the present study was to obtain insights into the physiological mechanisms underlying phenotypic variation in mastitis susceptibility, which might help identify molecular pathways and genes affecting mastitis susceptibility due to the SCS-BTA18-QTL using a combined approach of holistic gene expression profiling of primary bovine mammary gland epithelial cells (pbMEC) sampled from heifers that inherited alternative QTL alleles. In a previous study, prepartum primiparous heifers with a genetic predisposition for low or high SCS after parturition [27] were selected using the molecular marker information known for BTA18. Quantitative Real-Time-PCR (qRT-PCR) was used to specifically investigate the mRNA expression profiles of 10 innate immune system key molecules after bacterial challenge of pbMEC [12]. The first results showed that the less susceptible animals that inherited the favorable SCS-BTA18-QTL allele 'Q' (referred to as SCS-BTA18-Q animals) had a significantly elevated mRNA expression of innate immune response genes like *TLR2, TNF-α, IL-1β, IL-6 *and *IL-8 *24 h after bacterial challenge in comparison to the more susceptible animals that inherited the unfavorable SCS-BTA18-QTL allele 'q' (referred to as SCS-BTA18-q animals). In the current study, we expanded the analysis to a holistic transcriptome analysis using the Affymetrix GeneChip Bovine Genome Array to characterize global differences in gene expression in response to pathogen challenge in pbMEC sampled from SCS-BTA18-Q and SCS-BTA18-q animals. By analyzing the respective expression data using the short time-series expression miner STEM [28,29], co-expression profiles and significantly affected Ingenuity canonical pathways were identified providing first insights into genetically determined molecular mechanisms affecting mastitis susceptibility due to the SCS-BTA18-QTL.

## Methods

### Selection of animals

Heifers with either high or low susceptibility to mastitis were selected from the entire German Holstein population comprising heifers born between February and September 2003, that were sired for first parturition in a time interval of six weeks between December 2004 and February 2005. The detailed selection strategy and phenotypes of selected heifers are described by Kühn et al. [27]. In brief, three sires were selected from the German Holstein population based on the discrepancy of their marker-assisted best linear unbiased prediction (MA-BLUP) breeding values for SCS for their alternative haplotypes in the telomeric region of BTA18. Daughters of the three sires and their dams were genotyped at five marker loci (BM7109, ILSTS002, BMS2639, BM2078, TGLA227) within the telomeric region of BTA18 as described in Xu et al. [17]. The most likely paternally inherited marker haplotypes and thus, indirectly, the inherited paternal QTL alleles were inferred, and eleven heifers were selected from the pool of daughters. Six heifers (three heifers of sire 1, two heifers of sire 2, one heifer of sire 3) were assumed to have inherited the paternal chromosomal region decreasing SCS (SCS-BTA18-Q) and five heifers (three heifers of sire 1 and one heifer of each sire 2 and sire 3, respectively) were assumed to have inherited the paternal chromosomal region increasing SCS (SCS-BTA18-q). Dams and dam sires of the heifers were preselected for high (low susceptible heifers) and low relative estimated breeding values (high susceptible heifers) to increase the probability that the heifers inherited also the corresponding SCS-BTA18-QTL allele from the dams.

All 11 heifers were born and raised on different ordinary dairy farms. The heifers were collected at the Leibniz Institute for Farm Animal Biology Dummerstorf (FBN), in August 2005 at least 12 weeks prior to calving. They were kept in a free stall barn in one group under identical environmental conditions regarding housing, feeding and milking regime. The husbandry conditions were in accordance with national guidelines for animal experiments and standard dairy farm practice without any intervention in the living animal. The experimental approach was approved by an institutional committee. All individuals were slaughtered according to protocols for certified European slaughterhouses under the federal control of an independent veterinarian. The somatic cell count of the experimental and non-experimental cows in the dairy herd at the FBN was routinely below 100,000 cells/mL indicating a high management level of udder health. At day 42 postpartum, the individuals were slaughtered and a post mortem investigation of the udder and the carcass was performed. All heifers had no clinical mastitis and milk samples did not give indication of bacterial infection at slaughter.

### Primary cell culture of mammary epithelial cells

Primary cell cultures from the mammary gland epithelium were established as described by Griesbeck-Zilich et al. [12]. Immediately after slaughter of the selected heifers, two samples were taken aseptically from the parenchyma of the left rear quarter of the udder. The samples were transferred into Hank's balanced salt solution supplemented with antibiotics (HBSS; Sigma-Aldrich, Munich, Germany), and the tissue was minced and blood as well as milk residues were flushed away. Thereafter, the cells were transferred to a digestion mix of 200 mL HBSS supplemented with antibiotics, 0.5 mg/mL collagenase IA, 0.4 mg/mL DNase type I and 0.5 mg/mL hyaluronidase (enzymes from Sigma-Aldrich, Munich, Germany). After incubation, the cells were separated from connective tissue and non-epithelial cell conglomerates by filtration and centrifugation. Cells were then resuspended in Dulbecco's modified Eagle's medium nutrient mixture F-12 Ham (DMEM/F12, Sigma-Aldrich, Munich, Germany) containing 10% FBS and 10 μl/mL ITS (0.5 mg/ml bovine insulin, 0.5 mg/mL apo-transferrin, 0.5 μg/mL sodium selenite; Sigma-Aldrich, Munich, Germany). The cells were incubated for 40 min (37°C, 5% CO_2_, and 90% humidity) until the fibroblasts had attached and epithelial cells could be isolated by decanting. The cells were cryopreserved at -80°C in 1 mL freezing medium containing DMEM/F12, 20% FBS, and 10% DMSO. In order to verify the epithelial origin of the cells, an immunocytochemical staining of cytoceratins characterizing this cell type was conducted randomly as described [30]. The predominant cell type was represented by epithelial cells (approximately 90 to 95%).

### Treatment of epithelial cells with mastitis pathogens

Pathogen challenge and cell culture were performed essentially as described by Griesbeck-Zilch et al. [12]. Heat-inactivated *S. aureus *M60 and *E. coli *isolates derived from bovine milk samples of mastitis affected udders were used for inoculation [31]. Epithelial cells were thawed and cultured (37°C, 5% CO_2_, and 90% humidity) in DMEM/F12 medium for two further passages. For pathogen challenge, they were seeded in three six-well tissue culture plates (Greiner bio-one, Frickenhausen, Germany), one plate for each animal and each time point (1, 6 and 24 h), at a concentration of 300,000 cells/well. Two wells in each plate were prepared for control and one for each *S. aureus *and *E. coli *treatment. At a confluence of about 70% on the second day after seeding, the medium was refreshed. According to Wellnitz et al. [31], 100 μL of bacterial-solution representing a multiplicity of infection of 10, was added. 100 μL PBS were used as control treatment for the un-inoculated control cells.

### RNA extraction and microarray hybridization

Cells were harvested 1, 6, and 24 h after pathogen challenge, and total RNA was extracted with the TriFast reagent as described in the manufacturer's protocol (PEQLAB Biotechnology GmbH, Erlangen, Germany). After DNaseI treatment, RNA was removed using the RNeasy Kit (Qiagen, Hilden, Germany). RNA was quantified using a NanoDrop ND-1000 spectrophotometer (NanoDrop, PEQLAB Biotechnology GmbH, Erlangen, Germany) and its integrity was checked by running 1 μg of RNA on a 1% agarose gel. Comparative expression profiling was performed using the GeneChip Bovine Genome Arrays (Affymetrix, St. Clara, USA) comprising 24,072 probe sets representing approximately 19,000 UniGene clusters. According to the recommendations for microarray hybridization (Affymetrix, St. Clara, USA), antisense biotinylated RNA was prepared with 2 μg of total RNA using the GeneChip 3'IVT Express kit (Affymetrix, St. Clara, USA). After hybridization, arrays were scanned using the GeneChip scanner 3000 (Affymetrix, St. Clara, USA). The quality of hybridization was assessed in all samples following the manufacturer's recommendations using Affymetrix Expression Console version 1.1 (Affymetrix, St. Clara, USA). Additionally, the R-statistical language (distribution 2.9.2) and the affy (version 1.22.1) and affyPlm (version 1.20.0) packages from the Bioconductor microarray suit [32] were used for supplemental quality control. A complete list of all arrays included in the analyses is given in Table 1. After quality control, nine chips of the SCS-BTA18-q group and two chips of the SCS-BTA18-Q group were removed, because of higher centered and larger spread boxes in NUSE (Normalized Unscaled Standard Error) plots and an elevated RNA degradation indicated by the 5' to 3' ratio of GAPDH-RNA. Due to lack of biological material, these chips could not be repeated. The microarray data are deposited at Gene Expression Omnibus database [33] (GEO: GSE24560).

**Table 1:** Summary of microarrays included in the analysis

SCS-BTA18-QTL allele	Control			*E. coli*			*S. aureus*		
	**1 h**	**6 h**	**24 h**	**1 h**	**6 h**	**24 h**	**1 h**	**6 h**	**24 h**

Q	6	5	6	6	6	5	6	6	6
q	3	3	4	5	5	4	4	4	4

Number of microarrays passing the quality control for each time point, each treatment (*E. coli, S. aureus *and control treatment) and each of the inherited SCS-BTA18-QTL alleles (SCS-BTA18-Q, SCS-BTA18-q).

### Microarray preprocessing

The R statistical language (distribution 2.9.2) was used for data preprocessing. Microarray raw data were preprocessed using the RMA algorithm [34] for background correction, normalization by quantile normalization and summary measures by median polish. The data were filtered for absent genes by applying the MAS5 algorithm implemented in the Bioconductor affy package (version 1.22.1) for detection of present calls. Thereafter, Affymetrix control probe sets were removed from the datasets. Annotations of the Affymetrix identifiers to human gene symbols are based on Hintermair [35] supplemented with additional information obtained from the NetAffx annotation provided by Affymetrix.

### Statistical analysis and bioinformatics

After preprocessing of the microarray raw data, the BioConductor package Limma (version 2.18.3) [36] was used to identify differentially expressed genes. Limma applies an empirical Bayes approach based on linear models to assess the probability of differentially expressed genes. In this study, a three factorial design considering genotype, treatment and time point as factors was analyzed. A variety of tests was performed to confirm the effects of the QTL allele on cell culture and inoculation and to survey the consistency between analyses that could have been affected by the low number of chips within and the difference in the number of chips between groups. Analysis 1 was performed to compare gene expression levels between time points separately for each combination of factors treatment (*S. aureus, E. coli *and control) and genotype (SCS-BTA18-q and SCS-BTA18-Q). Analysis 2 was used to investigate differences in gene expression levels at time points between inoculated and control cells separately for each combination of factors genotype (SCS-BTA18-q and SCS-BTA18-Q) and pathogen (*S. aureus *and *E. coli*). Analysis 3 was performed to investigate differences in gene expression levels between time points for each fold change obtained between inoculated cells and control cells at time points (Analysis 2) separately for each combination of factors genotype (SCS-BTA18-q and SCS-BTA18-Q) and pathogen (*S. aureus *and *E. coli*), respectively. All investigated comparisons are listed in Table 2.

**Table 2:** Comparisons performed using Limma

Analyses	Comparison	Factors
Analysis 1	24 h - 1 h	treatment X genotype
	24 h - 6 h	
	6 h - 1 h	
Analysis 2	inoculated - control 24 h	pathogen X genotype
	inoculated - control 6 h	
	inoculated - control 1 h	
Analysis 3	(inoculated - control 24 h) - (inoculated - control 1 h)	pathogen X genotype
	(inoculated - control 24 h) - (inoculated - control 6 h)	
	(inoculated - control 6 h) - (inoculated - control 1 h)	

Summary of comparisons made in each of the three analyses; all analyses were performed separately for each combination of factors: genotype (SCS-BTA18-Q, SCS-BTA18-q) and treatment (*E. coli, S. aureus *and control treatment) in Analysis 1 or genotype (SCS-BTA18-Q, SCS-BTA18-q) and pathogen (*E. coli, S. aureus*) in Analysis 2 and Analysis 3.

Due to the low number of samples within groups and the difference in the number of samples between groups, a decreased power of the statistical analyses was expected. This problem is evident mainly in Analysis 3, because of the high number of tests in addition to the moderate number of factors and low numbers of samples. Analysis 3 was focused on the analysis of genes predominantly affected by pathogen challenge. Therefore, only genes with a minimum expression change of log_2 _fc ≥ 0.75 during time-course were considered. A fold change threshold was applied in order to include in the co-expression analysis, only the genes, showing elevated expression changes during time-course. With the log_2 _fc ≥ 0.75 a moderate fold change filter was applied [37]. The significance of co-expression was then assessed by applying the clustering algorithm implemented in the short time-series expression miner STEM (version 1.3.6) [28,29] for co-expression profiling and a subsequent comparison of the number of genes assigned to a specific co-expression profile model to the expected number of genes assigned to the co-expression profile model quantified by permutation. Because no expression profiling was performed at time point zero and control cells and inoculated cells derived from the same cell culture, no differences regarding gene expression between the inoculated and control cells were expected at time point zero. Hence, the 'no normalization/add 0' option was selected in STEM in Analysis 3 and all expression values at time point zero were set to zero to enable the co-expression profiling to include changes in gene expression levels in the first hour after bacterial challenge. The STEM clustering method [28] was chosen, and the maximum number of profiles was set to the default value of 50 considering a maximum unit change of 2 between profiles.

Contrary to Analysis 3, in Analysis 1 and Analysis 2 the moderated t-test statistics implemented in Limma considering a stringent significance threshold of an FDR adjusted p-value of q ≤ 0.05 were applied. Additionally, a fold change criterion was not applied in these analyses to monitor all significant expression changes due to cell culture or inoculation. For the biological interpretation of the data, significantly differentially (Analysis 1 and Analysis 2) and co-expressed (Analysis 3) genes were further analyzed using the Ingenuity Pathway Analysis 8.8 [38]. In addition, to compare and visualize gene expression levels, the hierarchical clustering method implemented in the MeV MultiExperiment Viewer v4.4 [39,40] was used.

## Results

### Effects of cell culture on gene expression in primary bovine mammary gland epithelial cells between cell culture time points of 1, 6 and 24 h

**Figure 1:**
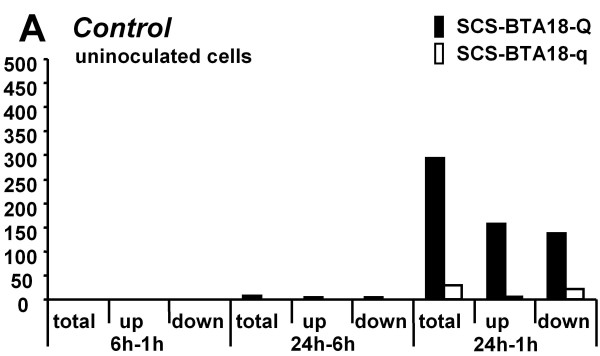
**Differentially expressed genes between time points 1, 6 and 24 h of cell culture**. Number of differentially genes (FDR adjusted p-value q ≤ 0.05) between time points 1, 6 and 24 h of cell culture for each of the inherited SCS-BTA18-QTL alleles, respectively.

To investigate the influence of cell culture on pbMEC sampled from SCS-BTA18-Q and SCS-BTA18-q animals, the differences in mRNA expression levels of control cells between time points 1, 6 and 24 h were analyzed separately for each SCS-BTA18-QTL allele (Figure 1). A first analysis of differentially expressed genes using the Ingenuity Pathway Analysis indicated that cellular and molecular processes affecting 'cell cycle' and 'cellular development' are regulated in response to cultivation after 24 h and that there is a difference in the response to cell culture between SCS-BTA18-Q and SCS-BTA18-q cells. Between time points 1 and 24 h, both, the cells derived from SCS-BTA18-Q animals and the cells derived from SCS-BTA18-q animals, showed substantial changes in gene expression. Whereas 293 genes were differentially expressed in SCS-BTA18-Q cells, only 28 genes were differentially expressed in the corresponding SCS-BTA18-q cells [see Additional file 1]. The difference in the number of differentially expressed genes between the two groups is partially related to the lower number of samples in the corresponding SCS-BTA18-q group (10 samples) compared to the SCS-BTA18-Q group (17 samples) affecting the power of the statistical analyses. However, only about 50% of the genes (14 genes) differentially expressed in the SCS-BTA18-q group were also found to be differentially expressed in the SCS-BTA18-Q group. Five of the six genes that were up-regulated towards time point 24 h (*LINS1, FBXL20, IRF2BP2, PHF13, DSEL*) and three of the top ten down-regulated genes (*NOL6, PDIA4, NEDD9*) in the SCS-BTA18-q cells showed the same direction of significant changes in expression levels in the SCS-BTA18-Q cells. Accordance in genes' regulation and differences in the genes regulated between SCS-BTA18-Q and SCS-BTA18-q cells suggested that common mechanisms were affected by cell culture but also that unique mechanisms were affected by the genotype. A subsequent functional analysis of the significantly differentially expressed genes associated with molecular and cellular functions related to 'cell cycle', 'cellular development' and 'cellular assembly and organization' was performed. In the SCS-BTA18-Q group, genes mainly associated with molecular and cellular functions affecting 'cell cycle progression' (*C15ORF63, FGF2, NEDD9, NOLC1, NRG1, PES1, PRMT5, RAN, SESN1, TBRG4*), 'rRNA processing' (*GEMIN4, NOLC1, NOP56, WDR43*) and the 'activation of gene expression' (*NEDD9, FGF2, NRG1, SMAD4*) were differentially expressed after 24 h of cell culture (Table 3). Although the number of genes in the SCS-BTA18-q group was low compared to the SCS-BTA18-Q group, single genes indicated that, at least in part the same molecular and cellular functions were affected in the SCS-BTA18-q group (Table 3). After 24 h of cell culture, genes associated with molecular and cellular functions involved in the 'regulation of the cell cycle' (*LMNA, NEDD9*), in the 'regulation of gene expression' (*NEDD9, MCRS1*) and in 'rRNA processing' (*GEMIN4*) were differentially expressed. Unique to the SCS-BTA18-q group was the decreased expression of *LMNA *and *FSCN1 *after 24 h of cell culture. Both genes are involved in several molecular and cellular functions including the 'organization of the actin cytoskeleton' and the 'differentiation and proliferation of epithelial cell lines' (*FSCN1*) as well as the 'nuclear assembly', the 'chromatin organization' and 'apoptosis signaling' (*LMNA*). Unique to SCS-BTA18-Q cells, was the differential expression of genes affecting molecular and cellular functions associated with 'small molecule biochemistry', 'nucleic acid metabolism' and 'carbohydrate metabolism'. In these cells, the down-regulation between time point 1 h and 24 h of *ERCC6, POLR2D, RAD23B*, genes that are involved in the nucleotide excision repair pathway, of RNA polymerase polypeptides *POLR1A, POLR1E, POLR2D, POLR3B *and *POLR3D*, genes that are involved in the pyrimidine and purine metabolisms, as well as the down-regulation of *GPI *and *TPI1 *that are involved in glycolysis and gluconeogenesis affirmed that the processes affected after 24 h of cell culture are mainly those important for cellular homeostasis.

**Table 3:** Molecular and cellular functions affected by cell culture

Top 5 categories of molecular and cellular functions	SCS-BTA18-Q		SCS-BTA18-q	
**Control cells SCS-BTA18-Q**	**p-values**	**Genes**	**p-values**	**Genes**

Cell cycle	1,98E-04	25	1,19E-02	2
Small molecule biochemistry	2,56E-04	19	---	---
Cellular development	7,60E-04	10	2,66E-03	1
Nucleic acid metabolism	7,60E-04	6	---	---
Carbohydrate metabolism	1,50E-03	9	---	---

**Top 5 categories of molecular and cellular functions**	**SCS-BTA18-Q**		**SCS-BTA18-q**	
**Control cells SCS-BTA18-q**	**p-values**	**Genes**	**p-values**	**Genes**

Cellular assembly and organization	1,28E-02	12	1,33E-03	2
Cellular function and maintenance	1,60E-02	6	1,33E-03	1
Cellular development	7,60E-04	10	2,66E-03	1
Cell morphology	2,48E-03	9	3,99E-03	1
Gene expression	3,68E-03	8	7,96E-03	2

Top five molecular and cellular functions affected in control cells after 24 h of cultivation; the molecular and cellular functional category and p-values as well as the number of involved genes are shown for SCS-BTA18-Q and SCS-BTA18-q cells.

### Effect of inoculation with heat inactivated *S. aureus *and *E. coli *pathogens on gene expression in primary bovine mammary gland cells between and at time points 1, 6 and 24 h of inoculation

**Figure 2:**
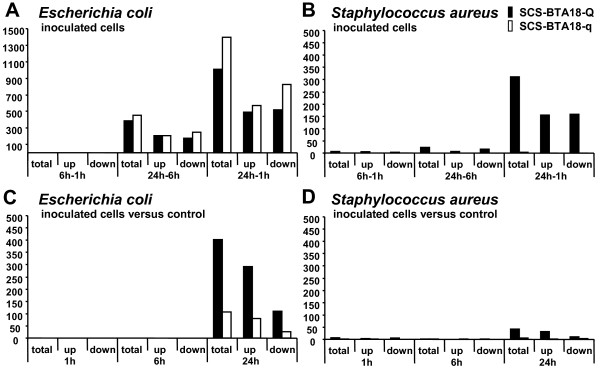
**Differentially expressed genes between and at time points 1, 6 and 24 h of bacterial challenge**. Number of differentially expressed genes (FDR adjusted p-value q ≤ 0.05) between time points, for each pathogen challenge and each of the inherited SCS-BTA18-QTL alleles as well as between inoculated cells and control cells at time points for each pathogen challenge and each of the inherited SCS-BTA18-QTL alleles; **A ***E. coli *inoculated cells; **B ***S. aureus *inoculated cells; **C ***E. coli *inoculated cells versus control; **D ***S. aureus *inoculated cells versus control.

Inoculation with either pathogen significantly affected gene expression in both SCS-BTA18-QTL groups. The most significant changes were observed when considering the whole time period between 1 h and 24 h of inoculation and the gene expression at time point 24 h between inoculated and control cells (Figure 2). Between time points 1 h and 24 h, *E. coli *inoculated cells showed a significantly higher number of differentially expressed genes (SCS-BTA18-Q: 1010 genes and SCS-BTA18-q: 1393 genes) in comparison to *S. aureus *inoculated cells (SCS-BTA18-Q: 312 genes and SCS-BTA18-q: four genes). Similarly, at time point 24 h, 402 and 43 genes were differentially expressed between *E. coli *and *S. aureus *inoculated cells and their respective un-inoculated control cells in the SCS-BTA18-Q group and 107 and five genes in the SCS-BTA18-q group, respectively. In comparison, the number of differentially expressed genes in inoculated cells between time points was higher than between inoculated and control cells at given time points suggesting that when analyzing between time points, a large proportion of the differentially expressed genes were affected by cell culture or by cumulative effects of cell culture and inoculation.

**Figure 3:**
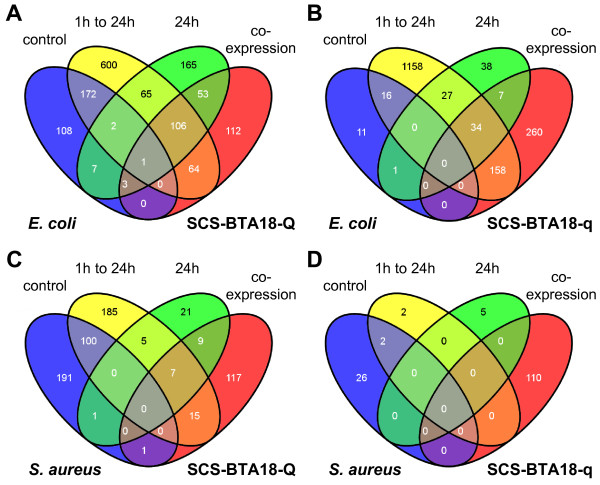
**Four-Set Venn diagrams comparing differentially expressed genes between analyses**. Comparison between significantly co-expressed genes at time point 24 h and significantly differentially expressed genes in control cells between time points 1 h and 24 h, in inoculated cells between time points 1 h and 24 h as well as between inoculated cells and control cells at time point 24 h for each pathogen and each QTL allele, respectively; **A **SCS-BTA18-Q cells inoculated with *E. coli*; **B **SCS-BTA18-q cells inoculated with *E. coli*; **C **SCS-BTA18-Q cells inoculated with *S. aureus*; **D **SCS-BTA18-q cells inoculated with *S. aureus.*

These observations are supported by the identified functional categories associated with the differentially expressed genes using Ingenuity Pathway Analysis. At time point 24 h, inoculated cells in comparison to control cells exhibited predominantly differentially expressed genes that were involved in molecular and cellular functions comprising 'hematological system development', 'inflammatory response', 'cell to cell signaling' and 'immune cell trafficking' (Table 4). These genes were exclusively regulated in inoculated cells but not in control cells during time-course (Figure 3). In addition, differentially expressed genes between time points 1 h and 24 h in both inoculated and control cells were significantly associated with molecular and cellular functions comprising 'cell cycle', 'cellular growth and proliferation', 'DNA replication, recombination and repair' and 'cell death' (Table 5). However, these differences were more pronounced in inoculated cells in comparison to the control cells. Furthermore, the number of genes assigned to each of the top five molecular and cellular function categories between time points 1 h and 24 h was higher in *E. coli *inoculated cells compared to *S. aureus *inoculated and control cells. These results indicated that cellular processes important for cellular homeostasis are more seriously affected by inoculation with *E. coli *than with *S. aureus*.

**Table 4:** Biological functions affected by inoculation solely

	*E. coli*				*S. aureus*	
**Top 5 categories of biological functions**	**SCS-BTA18-Q**		**SCS-BTA18-q**		**SCS-BTA18-Q**	

***E. coli *versus control SCS-BTA18-Q**	**p-values**	**Genes**	**p-values**	**Genes**	**p-values**	**Genes**

Cell death	1,08E-13	96	8,30E-05	18	5,00E-05	14
Cell-to-cell signaling and interaction	3,60E-13	51	5,35E-03	12	2,45E-04	7
Hematological system development and function	3,60E-13	53	7,87E-05	14	8,09E-05	8
Immune cell trafficking	3,60E-13	34	8,62E-04	7	8,09E-05	5
Tissue development	3,60E-13	38	6,51E-03	5	3,49E-03	4

	***E. coli***				***S. aureus***	

**Top 5 categories of biological functions**	**SCS-BTA18-Q**		**SCS-BTA18-q**		**SCS-BTA18-Q**	

***E. coli *versus control SCS-BTA18-q**	**p-values**	**Genes**	**p-values**	**Genes**	**p-values**	**Genes**

Hematological system development and function	3,60E-13	53	7,87E-05	14	8,09E-05	8
Hematopoesis	8,87E-07	27	7,87E-05	9	1,03E-04	5
Cell death	1,08E-13	96	8,30E-05	18	5,00E-05	14
Cellular development	1,67E-07	56	9,58E-05	11	1,52E-05	9
Gene expression	3,39E-11	81	1,25E-04	10	1,11E-03	10

	***E. coli***				***S. aureus***	

**Top 5 categories of biological functions**	**SCS-BTA18-Q**		**SCS-BTA18-q**		**SCS-BTA18-Q**	

***S. aureus *versus control SCS-BTA18-Q**	**p-values**	**Genes**	**p-values**	**Genes**	**p-values**	**Genes**

Inflammatory response	4,38E-11	51	3,63E-03	11	8,30E-06	7
Cellular development	1,67E-07	56	9,58E-05	11	1,52E-05	9
Cellular growth and proliferation	9,89E-12	112	5,35E-03	18	1,52E-05	15
Tissue morphology	5,40E-05	13	6,51E-03	2	4,12E-05	3
Cell death	1,08E-13	96	8,30E-05	18	5,00E-05	15

Top 5 biological functions affected between inoculated SCS-BTA18-Q and SCS-BTA18-q cells and respective control cells; the functional category, p-values and the number of genes are shown for *E. coli *inoculated SCS-BTA18-Q and SCS-BTA18-q cells and *S. aureus *inoculated SCS-BTA18-Q cells; the categories are ranked by p-values of the SCS-BTA18-Q and SCS-BTA18-q cells, respectively and related p-values and the number of involved genes are shown for the alternative QTL allele and pathogen; for *S. aureus *inoculated SCS-BTA18-q cells the number of significantly differentially expressed genes was to low to perform a reliable investigation of associated molecular and cellular functions.

**Table 5:** Molecular and cellular functions affected by inoculation and cell culture

	*E. coli *inoculated				Un-inoculated control			
**Top 5 categories of molecular and cellular functions**	**SCS-BTA18-Q**		**SCS-BTA18-q**		**SCS-BTA18-Q**		**SCS-BTA18-q**	

***E. coli *inoculated** **SCS-BTA18-Q cells**	**p-values**	**Genes**	**p-values**	**Genes**	**p-values**	**Genes**	**p-values**	**Genes**

Cell cycle	2,98E-18	120	2,26E-25	164	1,98E-04	25	1,19E-02	2
Cellular growth and proliferation	1,10E-10	215	2,56E-11	279	1,28E-02	10	3,28E-02	2
Cellular assembly and organization	9,30E-10	59	2,14E-10	70	1,28E-02	12	1,33E-03	2
DNA replication, recombination and repair	9,30E-10	96	2,14E-10	154	3,18E-02	5	---	---
RNA-post-transcriptional modification	3,70E-06	39	9,50E-06	42	1,58E-03	11	4,56E-02	1

	***E. coli *inoculated**				**Un-inoculated control**			

**Top 5 categories of molecular and cellular functions**	**SCS-BTA18-Q**		**SCS-BTA18-q**		**SCS-BTA18-Q**		**SCS-BTA18-q**	

***E. coli *inoculated** **SCS-BTA18-q cells**	**p-values**	**Genes**	**p-values**	**Genes**	**p-values**	**Genes**	**p-values**	**Genes**

Cell cycle	2,98E-18	120	2,26E-25	164	1,98E-04	25	1,19E-02	2

Cellular growth and proliferation	1,10E-10	215	2,56E-11	279	1,28E-02	10	3,28E-02	2

Cellular assembly and organization	9,30E-10	59	2,14E-10	70	1,28E-02	12	1,33E-03	2

DNA replication, recombination and repair	9,30E-10	96	2,14E-10	154	3,18E-02	5	---	---

Cell death	7,33E-06	153	2,27E-10	227	6,73E-03	8	---	---

	***S. aureus *inoculated**		***E. coli *inoculated**				**Un-inoculated control**	

**Top 5 categories of molecular and cellular functions**	**SCS-BTA18-Q**		**SCS-BTA18-Q**		**SCS-BTA18-q**		**SCS-BTA18-Q**	

***S. aureus *inoculated** **SCS-BTA18-Q cells**	**p-values**	**Genes**	**p-values**	**Genes**	**p-values**	**Genes**	**p-values**	**Genes**

Cellular assembly and organization	6,54E-05	14	9,30E-10	59	2,14E-10	70	1,28E-02	12
Cell death	2,03E-04	33	7,33E-06	153	2,27E-10	227	6,73E-03	8
DNA replication, recombination and repair	2,61E-04	20	9,30E-10	96	2,14E-10	154	3,18E-02	5
Nucleic acid metabolism	2,61E-04	9	3,54E-03	4	4,61E-03	14	7,60E-04	6
Small molecule biochemistry	2,61E-04	18	7,60E-05	53	3,80E-03	26	2,56E-04	19

Top five molecular and cellular functions affected between time points 1 h and 24 h in SCS-BTA18-Q and SCS-BTA18-q cells by inoculation; the molecular and cellular functional category, p-values and the number of involved genes are shown for *E. coli *inoculated SCS-BTA18-Q and SCS-BTA18-q cells and *S. aureus *inoculated SCS-BTA18-Q cells, as well as for the control cells; the categories are ranked by p-values of the SCS-BTA18-Q and SCS-BTA18-q cells, respectively, and related p-values and the number of involved genes are shown for the alternative QTL allele and the un-inoculated control cells; for *S. aureus *inoculated SCS-BTA18-q cells the number of significantly differentially expressed genes was to low to perform a reliable investigation of associated molecular and cellular functions; hence, for SCS-BTA18-Q cells additionally the related p-values and the number of involved genes are shown for *E. coli *inoculated cells and for un-inoculated SCS-BTA18-Q control cells.

However, *S. aureus *inoculation resulted in an elevated number of differentially expressed genes assigned to the functional categories 'cell death' and 'DNA replication, recombination and repair' in SCS-BTA18-Q cells between time points 1 h and 24 h in comparison to control cells indicating that *S. aureus *inoculation affected processes important for cellular homeostasis more seriously than cell culture. This analysis was done on SCS-BTA18-Q cells only, because the number of significantly differentially expressed genes was too low in *S. aureus *inoculated SCS-BTA18-q cells to perform a reliable investigation of associated molecular and cellular functions.

Nevertheless, the observed effects of cell culture and pathogen challenge on gene expression in pbMEC clearly indicate the suitability of the established *in vitro *system to study the cellular and molecular response to effects of endogenous and exogenous factors like effects of the SCS-BTA18-QTL alleles.

### Effects of SCS-BTA18-QTL alleles on the response to pathogen challenge: co-expression profiling and Ingenuity Pathway analysis

**Figure 4:**
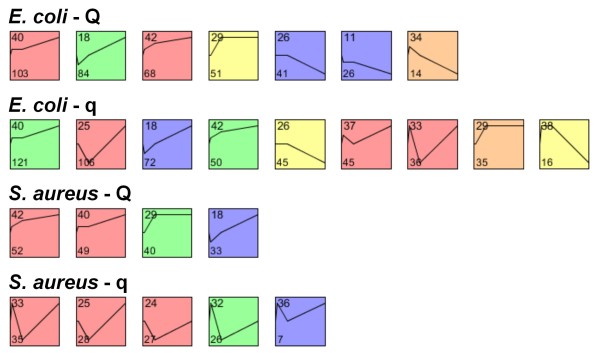
**Significant co-expression profiles**. Significantly enriched co-expression profiles clustered by the short time-series expression miner (STEM); profiles are ordered based on the p-value significance of the number of genes assigned to the co-expression profile versus the number of genes expected quantified by permutation; only significantly enriched profiles are shown; each square represents one probe level model; the line within the square represents the changes in the expression level during time-course between inoculated and control cells; in the upper left corner the number of the profile and in the lower left corner the number of assigned genes are shown; colors indicate similar profiles within each analysis.

To study the effects of SCS-BTA18-QTL alleles on the response to pathogen challenge, the non-random co-expression of genes was assessed by applying a permutation test to overcome the difficulty in assessing an appropriate significance level enabling an unbiased comparison between SCS-BTA18-Q and SCS-BTA18-q cells. The co-expression profiles that were significantly enriched for genes showing a similar expression profile during time-course are shown in Figure 4. A table of genes including log fold changes for significantly enriched profiles is given in additional file 2 [see Additional file 2]. Most of the 14 different significant profiles (10 profiles) indicated an up-regulation of genes towards time point 24 h. Remarkably, all of the profiles up-regulated after 24 h in SCS-BTA18-Q cells showed an early and linear up-regulation of co-expressed genes, whereas all profiles in SCS-BTA18-q cells inoculated with *S. aureus *and in part in those with *E. coli *(profiles 25 and 33) showed a delayed up-regulation of genes after 6 h of inoculation (Figure 4). These different expression profiles are characterized by genes mainly associated with the functional categories 'cell death' (*ADM, AGR2, BIRC3, BNIP3, CASP3, CASP4, CCL5, DDX58, DUSP1, FLI1, IER3, IFI16, LMO2, NFKBIA, NOS2, PTGS2, STK38, USP18*), 'complement system' (*C1R, C1S *and *CFH*) and 'chemotaxis of neutrophils' (*CCL5 *and *CXCL2*).

**Figure 5:**
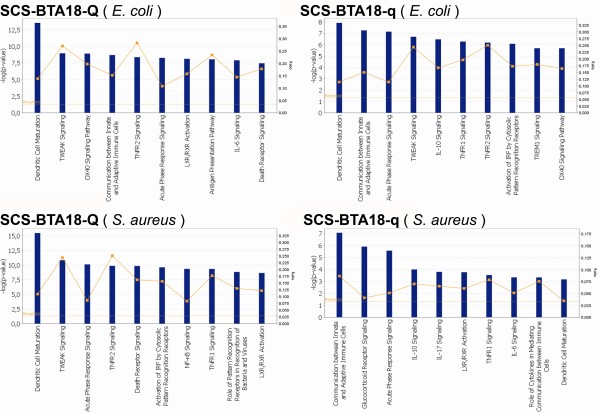
**Overview canonical pathways**. Ingenuity canonical pathways affected during time-course between inoculated and control cells in SCS-BTA18-Q and SCS-BTA18-q cells inoculated with *E. coli *and *S. aureus*, respectively; blue bars indicate p-value significance and the orange threshold line indicates the p ≤ 0.05 significance thresholds; orange squares and lines indicate the ratio of genes found to be involved in the specific pathway to the overall number of genes involved in that pathway.

To obtain a more detailed view of pathways affected by the SCS-BTA18-QTL alleles, all of the significantly co-expressed genes were included in the Ingenuity Pathway Analysis for a biological interpretation of the data. In a first step, Ingenuity canonical pathways were investigated. An overview of significantly affected canonical pathways is given in Figure 5. Comparing canonical pathways affected in SCS-BTA18-Q and SCS-BTA18-q cells as well as in *E. coli *and *S. aureus *inoculated cells indicated that most of the significant canonical pathways were affected in both SCS-BTA18-QTL groups. However, the different ranks of canonical pathways based on p-values and the number of co-regulated genes within pathways between SCS-BTA18-Q and SCS-BTA18-q cells indicated that there are pathogen-specific differences in the response to inoculation between both SCS-BTA18-QTL alleles. In SCS-BTA18-q cells, the most significantly affected canonical pathways were 'communication between innate and adaptive immune cells' as well as 'acute phase response signaling', whereas in SCS-BTA18-Q cells 'dentritic cell maturation' and 'TWEAK signaling' were predominantly affected. 'Dentritic cell maturation' and 'acute phase response signaling' were two of the most significantly affected pathways for both SCS-BTA18-QTL alleles and both pathogen challenges. However, *E. coli *inoculated SCS-BTA18-Q cells showed a significantly higher number of differentially expressed genes in comparison to SCS-BTA18-q cells and both *S. aureus *inoculated cells (Table 6). The most prominent genes associated with 'dendritic cell maturation' belonged to the major histocompatibility complex class 2 molecules namely *HLA-DMA, HLA-DMB, HLA-DQA1, HLA-DQB1, HLA-DRA *and *HLA-DRB1*, to genes involved in NF-kappaB signaling, namely *NFKB1, NFKB2, NFKBIA, NFKBIB, NFKBIE, IKBKE *and *RELB *and to the Interleukin 1 cytokine family members, namely *IL1A, IL1B, IL1F6 *and *IL1RN*. Genes like *CD40, NFKBIA, NFKBIZ, IKBKE, TLR2, IL1A *and *IL1B *that are also involved in 'dendritic cell maturation' showed an earlier and superior pathogen specific up-regulation in SCS-BTA18-Q cells in comparison to the SCS-BTA18-q cells. In contrast, genes of the 'acute phase response signaling' pathway such as *SAA3P, IL6 *and *NFKB2 *showed an earlier and higher up-regulation after inoculation with both pathogens in SCS-BTA18-Q cells in comparison to SCS-BTA18-q cells (Figure 4, profiles 40 and 42).

**Table 6:** Gene table canonical pathways

QTL allele	Pathogen	Canonical pathway	Genes
SCS-BTA18-Q	*E.coli*	Dendritic cell maturation	*HLA-DMA, IL1A, ICAM1, RELB, NFKBIE, IL1F6, HLA-DQA1, HLA-DRB1, LTB, HLA-DMB, IKBKE, IL6, NFKB2, NFKB1, TLR2, HLA-DQB1, NFKBIA, HLA-A, CD40, IL1RN, HLA-DRA, COL10A1, IL1B, NFKBIB*
		Acute phase response signaling	*IL1A, SAA3P, APOA1, RRAS, NFKBIE, C1S, IL1F6, IKBKE, IL6, NFKB2, NFKB1, HMOX2, C1R, SOD2, NFKBIA, IL1RN, CFB, IL1B, NFKBIB*
SCS-BTA18-q	*E.coli*	Dendritic cell maturation	*HLA-DMA, IL1A, ICAM1, NFKBIE, IL1F6, HLA-DRB1, LTB, IKBKE, HLA-DMB, IL6, NFKB2, NFKB1, TLR2, HLA-DQB1, NFKBIA, HLA-A, CD40, IL1RN, HLA-DRA, IL1B*
		Acute phase response signaling	*IL1A, SAA3P, C3, NFKBIE, C1S, SOCS2, IL1F6, IKBKE, IL6, NFKB2, NFKB1, HMOX2, C1R, HMOX1, SOD2, NFKBIA, IL1RN, CFB, IL1B, C2*
SCS-BTA18-Q	*S. aureus*	Dendritic cell maturation	*IL1A, ICAM1, RELB, NFKBIE, HLA-DQA1, PIK3R5, HLA-DRB1, LTB, IKBKE, IL6, NFKB2, NFKB1, TLR2, HLA-DQB1, NFKBIA, CD40, IL1RN, HLA-DRA, IL1B*
		Acute phase response signaling	*SOCS1, IL1A, SAA3P, APOA1, NFKBIE, IKBKE, NFKB2, IL6, NFKB1, HMOX2, NFKBIA, SOD2, IL1RN, CFB, IL1B*
SCS-BTA18-q	*S. aureus*	Acute phase response signaling	*C1R, HMOX1, IL1A, NFKBIA, C1S, IL1F6, IL6, FGG, CRABP1*
		Dendritic cell maturation	*HLA-DQB1, IL1A, NFKBIA, IL1F6, CD83, IL6*

Summary of genes associated with Ingenuity canonical pathways 'dendritic cell maturation' and 'acute phase response signaling' in *S. aureus *and *E. coli *inoculated SCS-BTA18-Q and SCS-BTA18-q cells, respectively.

**Figure 6:**
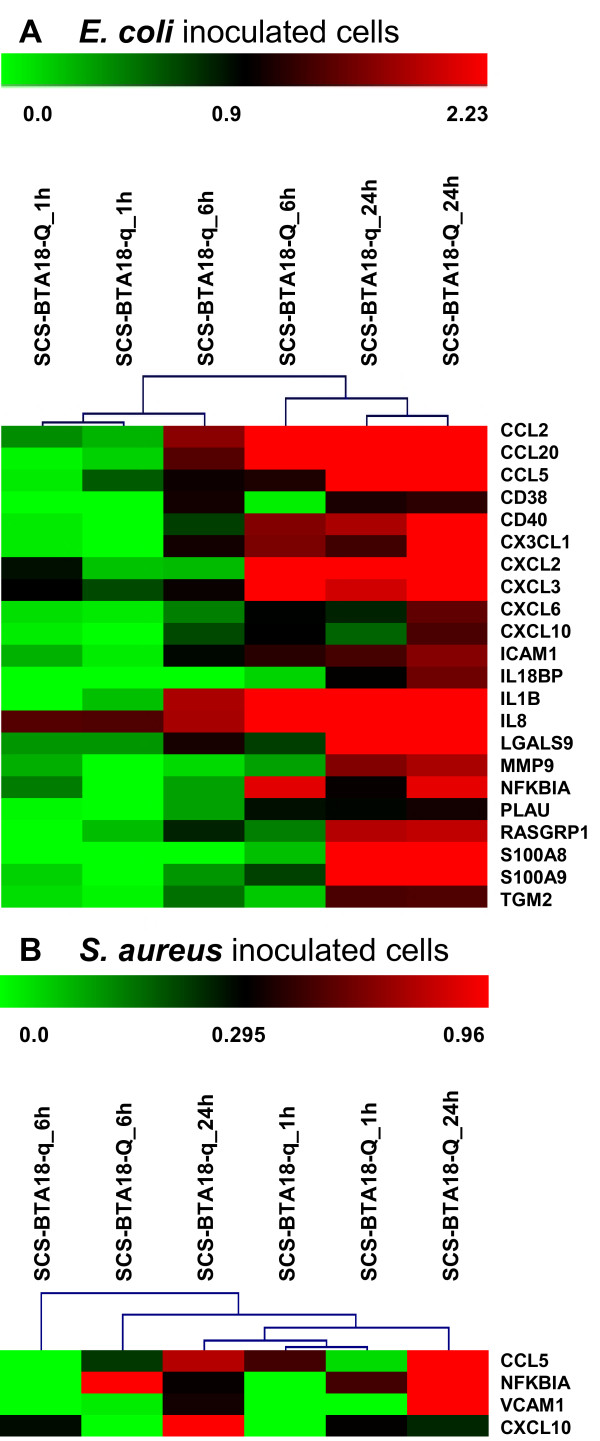
**Hierarchical clustering of genes associated with leukocyte migration**. Hierarchical clustering of expression data obtained for significantly co-expressed genes in SCS-BTA18-Q and SCS-BTA18-q cells associated with the Ingenuity functional category 'immune cell trafficking' that are involved in the migration of leucocytes; **A ***E. coli *inoculated cells; **B ***S. aureus *inoculated cells; heat map visualizes changes in gene expression levels between inoculated and control cells at time points; the log_2 _fold change ranges are shown at the upper bars.

In addition, we investigated genes that are involved in the 'migration of leukocytes' associated with the physiological system development and function category 'immune cell trafficking', which was significantly regulated by both pathogen challenges and SCS-BTA18-QTL alleles (Table 4). This was done, because genes involved in leukocyte migration could have a large effect on pathogen clearance and on SCS. Here, we applied the hierarchical clustering method implemented in the MeV MultiExperiment Viewer v4.4 [39,40] to compare and visualize gene expression between SCS-BTA18-Q and SCS-BTA18-q cells after pathogen challenge (Figure 6). In both challenges, SCS-BTA18-Q cells showed a faster response in comparison to SCS-BTA18-q cells. Thus, after inoculation with both pathogens cytokines showed an earlier and faster up-regulation towards time point 24 h in SCS-BTA18-Q cells in comparison to SCS-BTA18-q cells. In addition, a substantial difference in the composition of cytokines up-regulated in response to *S. aureus *challenge between SCS-BTA18-Q and SCS-BTA18-q cells was observed (Table 7). In *E. coli *inoculated SCS-BTA18-Q cells, 22 of the 29 genes affecting leukocyte migration were also up-regulated in SCS-BTA18-q cells, whereas in *S. aureus *inoculated SCS-BTA18-Q cells, only four of the 18 genes significantly co-expressed were also up-regulated in SCS-BTA18-q cells (Table 7). In particular, the elevated expression level of *CXCL2 *and *CXCL3 *1 h after inoculation with *E. coli *showed that SCS-BTA18-Q cells can initiate an early response to inoculation by the up-regulation of cytokines involved in the inflammatory response and in chemotaxis in comparison to SCS-BTA18-q cells. Furthermore, the hierarchical clustering indicated that the up-regulation of genes involved in leukocyte migration already occurred preferentially in the first 6 h in SCS-BTA18-Q cells inoculated with *E. coli*, whereas in SCS-BTA18-q cells several genes (*CXCL2, NFKBIA*) did not show an elevated expression until 24 h after inoculation. The difference in the regulation of genes during time-course between SCS-BTA18-q and SCS-BTA18-Q cells was more pronounced in *S. aureus *than in *E. coli *inoculated cells, which could be attributed to the delayed up-regulation of genes 6 h after inoculation in SCS-BTA18-q (Figure 4). Thus, after *S. aureus *inoculation, SCS-BTA18-Q cells showed a continuous up-regulation towards time point 24 h in the corresponding hierarchical clustering analysis, whereas the expression data of SCS-BTA18-q cells at time point 24 h and of both SCS-BTA18-Q and SCS-BTA18-q cells at time point 1 h were clustered together. In summary, SCS-BTA18-q showed a less distinct and delayed response to pathogen challenge in comparison to SCS-BTA18-Q in both *S. aureus *and *E. coli *inoculated cells, and *E. coli *inoculated cells triggered a faster and more distinctive response to pathogen challenge than *S. aureus *did. To identify potential candidate genes underlying the SCS-BTA18-QTL, a combined survey considering differentially expressed and positional candidate genes was performed, indicating a single gene, *v-rel reticuloendotheliosis viral oncogen homolog B *(*RELB*) to be differentially expressed after inoculation in SCS-BTA18-Q, but not SCS-BTA18-q cells and to be located in the vicinity of the SCS-BTA18-QTL.

**Table 7:** Gene table functional category immune cell trafficking

QTL allele	Pathogen	Category	Functional annotation	Genes
SCS-BTA18-Q	*E. coli*	Immune cell trafficking	Migration of leukocytes	*CCL2, CCL5, CCL20, CCL28, CD38, CD40, CSF1, CX3CL1, CXCL2, CXCL3, CXCL6, CXCL10, CXCL14, FAS, ICAM1, IL8, IL18BP, IL1B, ITGAV, LGALS9, MMP9, NFKBIA, PLAU, RASGRP1, S100A8, S100A9, TGM2, TNFRSF6B, VCAM1*
SCS-BTA18-Q	*S. aureus*	Immune cell trafficking	Migration of leukocytes	*CCL2, CCL5, CCL20, CD40, CXCL6, CXCL10, EDN1, FAS, ICAM1, IL1B, LGALS9, MMP9, NFKBIA, PLAU, RASGRP1, S100A8, S100A9, VCAM1*
SCS-BTA18-q	*E. coli*	Immune cell trafficking	Migration of leukocytes	*C3, CCL2, CCL5, CCL20, CD38, CD40, CX3CL1, CXCL2, CXCL3, CXCL6, CXCL10, CXCR4, CXCR7, EDN1, ICAM1, IL8, IL18BP, IL1B, ITGA5, LGALS9, MMP9, NFKBIA, PLAU, RASGRP1, S100A8, S100A9, TGM2*
SCS-BTA18-q	*S. aureus*	Immune cell trafficking	Migration of leukocytes	*CCL5, CXCL2, CXCL3, CXCL10, CXCR4, CXCR7, IL8, IL18BP, NFKBIA, VCAM1*

Summary of differentially expressed genes associated with Ingenuity functional category 'immune cell trafficking' that are involved in the migration of leukocytes in *S. aureus *and *E. coli *inoculated SCS-BTA18-Q and SCS-BTA18-q cells, respectively.

## Discussion

In this study, some first insights into the molecular mechanisms of the response to bacterial challenge of mammary gland epithelial cells sampled from half-sib heifers marker selected for alternative SCS-BTA18-QTL alleles were drawn from a holistic transcriptome analysis. The main findings of this study were firstly, that both, cell culture and inoculation, triggered significant changes in gene expression of mammary epithelial cells *in vitro*. Secondly, inoculation with heat-inactivated *E. coli *induced a stronger immune response compared to inoculation with heat-inactivated *S. aureus *within the first 24 h. Thirdly, both, cells sampled from animals inheriting the favorable QTL allele 'Q' and cells sampled from animals inheriting the unfavorable QTL allele 'q' could activate immune response mechanisms after bacterial challenge *in vitro*, but there was a delayed and weaker response in SCS-BTA18-q cells.

Numerous studies have shown that mammary epithelial cells play a crucial role in the response to invading pathogens in the mammary gland [31,41,42], and several studies have used primary bovine mammary gland epithelial cell cultures to investigate common mechanisms of immune response in mammary epithelial cells in response to mastitis pathogens *in vitro *[8,30,43-45]. The differences in the response to bacterial challenge of mammary epithelial cells *in vivo *and *in vitro *have been partially characterized by [8,45], indicating that only a subset of the genes involved in the immune response *in vivo *are regulated in mammary epithelial cells *in vitro *and that there is a difference in the time-course of the response. It has been suggested that these differences could be related to missing virulence factors of heat-inactivated pathogens that were used in *in vitro *experiments compared to the active pathogens used in *in vivo *models and to other immune cells regulating the gene expression of mammary epithelial cell *in vivo *[8,45]. However, most of the studies showed that primary bovine mammary gland epithelial cells can trigger an immune response after bacterial challenge or inoculation with bacterial cell wall components [8,30,44,46]. In particular, an induced expression was observed for inflammatory chemokines like *IL-8, CCL20, CCL5 *and *CCL2 *that are involved in neutrophile, lymphocyte and monocyte recruitment as well as for genes with antimicrobial activity such as *S100A9 *and *S100A12 *and for acute phase proteins like *SAA3P *and *HP *[8,30,44,45]. Our aim was to survey, if the present established *in vitro *test system is suitable to investigate molecular mechanisms regulated in the response to bacterial challenge and if there are differences in the response to pathogen challenge that are related to the different genetic predisposition of the animals.

### Effects of cell culture on gene expression in primary bovine mammary gland epithelial cells

Firstly, our study demonstrated that the cells sampled from SCS-BTA18-Q and SCS-BTA18-q animals responded to cell culture and that processes mainly involved in 'cell cycle' and 'cellular development' were affected by cell culture after 24 h. In particular, the down-regulation of genes associated with molecular and cellular function like 'small molecule biochemistry', 'nucleic acid metabolism' and 'carbohydrate metabolism' in SCS-BTA18-Q cells, comprising genes involved in the nucleotide excision repair pathway, in pyrimidine and purine metabolisms as well as glycolysis and gluconeogenesis, indicated that processes essential for cell survivability are down-regulated during culture. The number of differentially expressed genes in SCS-BTA18-q cells after 24 h of culture was low compared to SCS-BTA18-Q cells. Correspondingly, the observed effects of cell culture were more pronounced in SCS-BTA18-Q cells, which could be in part attributed to the lower number of samples in the SCS-BTA18-q group. However, the high coincidence of the top up- and down-regulated genes between SCS-BTA18-Q and SCS-BTA18-q control cells, the observed distinct response of pathogen challenged SCS-BTA18-q- after 24 h, and the distinct response of SCS-BTA18-Q and SCS-BTA18-q cells during time-course, both, after cell culture and inoculation, indicate that the limited number of differentially expressed genes in the control SCS-BTA18-q cells in comparison with the control SCS-BTA18-Q cells reflects well the observed delayed and weaker response after challenge with pathogens.

### Effect of inoculation with heat-inactivated *S. aureus *and *E. coli *pathogens on gene expression in primary bovine mammary gland cells

The response to inoculation with heat-inactivated *E. coli *and *S. aureus *showed pathogen specific effects on the gene expression in pbMEC with an elevated number of significantly differentially expressed genes observed for *E. coli *inoculated cells compared to *S. aureus *inoculated cells within the first 24 h. A faster and more pronounced immune response to *E. coli *in comparison to *S. aureus *is also known from other studies investigating response mechanisms of the mammary gland *in vitro *and *in vivo *[45-48]. Different analyses were performed to characterize the response of the mammary gland epithelial cells to bacterial challenge in this study. All three analyses, i.e. analysis between time points, analysis at time points between inoculated and un-inoculated cells and co-expression analysis showed that inoculation of cells sampled from SCS-BTA18-Q and SCS-BTA18-q animals with *E. coli *stimulated the expression of genes involved in the 'migration of leukocytes' as well as in canonical pathways associated with 'dendritic cell maturation' and 'acute phase response'. *SAA3, S100A9, IL-1β, CCL5, MX2 *and *BF *were some of the genes stimulated 24 h after *E. coli *inoculation, that have previously been shown to be significantly up-regulated in response to *E. coli *challenge in pbMEC [8]. Essentially, these results confirmed the results obtained in the analyses of innate immune system key molecules by RT PCR [12] investigating the same cells. After *E. coli *challenge, the microarray results at time point 24 h were in agreement to the respective results obtained with RT PCR [12]. *TLR 2, IL-1β, IL-6, IL-8, LTF *and *C3 *showed a higher expression in cells sampled from SCS-BTA18-Q animals compared to cells sampled from SCS-BTA18-q animals. In the *S. aureus *inoculated cells, all of these genes showed a higher expression level 24 h after inoculation in SCS-BTA18-Q cells compared to SCS-BTA18-q cells in the microarray analyses, hence, fully confirming the previous results obtained by RT PCR at time point 24 h [12]. Observed effects of cell culture and pathogen challenge on gene expression in pbMEC clearly indicated that the established *in vitro *system is suitable to study the cellular and molecular response to effects of endogenous and exogenous factors like effects of the SCS-BTA18-QTL alleles. This is in agreement with an ovine animal model [49], which also used sheep mammary gland epithelial cells to identify molecular mechanisms that are affected by selection for high and low SCS in two divergent lines of sheep selected by applying a selection strategy based on conventional breeding values.

### Effects of SCS-BTA18-QTL alleles on the response to pathogen challenge

In the present study, cells sampled from SCS-BTA18-Q animals exhibited corresponding changes in gene expression after pathogen challenge in accordance to other studies investigating molecular mechanisms of immune response in mammary gland epithelial cells [6,8,47]. In contrast, cells sampled from animals inheriting the SCS-BTA18-q allele showed a delayed and less distinct immune response associated gene expression to pathogen challenge. The comparison of genes affecting leukocyte migration between SCS-BTA18-Q and SCS-BTA18-q cells clearly showed that SCS-BTA18-Q cells triggered a faster response to *E. coli *inoculation indicated by the early and linear up-regulation of *CCL2, CCL20, CXCL2, CXCL3, IL1B, IL-8 *and *NFKBIA*. These genes are important for the inflammatory response and for the recruitment of monocytes, lymphocytes, neutrophils and basophils, which in turn are essential for a fast pathogen clearance [41,50]. On the contrary, cells from animals inheriting the SCS-BTA18-q allele showed a delayed up-regulation of those genes towards time point 24 h in response to *E. coli *challenge. The observed early and linear up-regulation of inflammatory chemokines after *E. coli *or *S. aureus *inoculation in SCS-BTA18-Q cells is in line to the earlier and higher up-regulation after pathogen challenge of *SAA3P, IL6 *and *NFKB2*, genes that are involved in 'acute phase response signaling'.

The differences observed between SCS-BTA18-Q and SCS-BTA18-q cells inoculated with *S. aureus *were more distinct than in *E. coli *inoculated cells. Whereas a high number of genes were regulated in common after *E. coli *challenge in SCS-BTA18-Q and SCS-BTA-q cells, only *CCL5, CXCL10, NFKBIA *and *VCAM1 *were in common and significantly regulated in SCS-BTA18-q and SCS-BTA18-Q cells after *S. aureus *challenge. In addition, the expression data of SCS-BTA18-q cells at time point 24 h clustered together with the expression data of SCS-BTA18-q and SCS-BTA18-Q cells at time point 1 h underlining a delayed response in SCS-BTA18-q cells.

Interestingly, by comparing the differentially expressed genes in SCS-BTA18-Q and SCS-BTA18-q cells a single gene located in the vicinity of the SCS-BTA18-QTL, *RELB*, was exclusively and significantly regulated in SCS-BTA18-Q cells. In a previous study we could identify the two-marker haplotype *BB710 - PVRL2_c.392G > A *within a 1 Mb range of the *RELB *locus as associated with SCS in the German Holstein Population [15]. *RELB *is involved in the non-canonical NF-kappaB signaling as part of the RelB/p52 complex [51]. Further analysis of genes involved in non-canonical NF-kappaB signaling also indicated that *CD40 *and *TNFSF13B *(*BAFF*), two receptors eliciting the non-canonical NF-kappaB signaling are significantly co-expressed in SCS-BTA18-Q cells as well as *NFKB2*, the gene encoding the p100 protein, which is processed into p52 to activate the RelB/p52 complex. The RelB/p52 complex is thought to be important in biological functions such as lymphoid organogenesis, B-cell survival and maturation and dendritic cell activation [51,52]. In addition, *RELB *deficient mice exhibited a multifocal, mixed inflammatory cell infiltration in several organs [53] and fibroblasts from *RELB *deficient mice, also indicated an important role of *RELB *as transcription suppressor limiting the expression of proinflammatory mediators [54]. This would be in line with the higher susceptibility of SCS-BTA18-q animals indicated by the higher SCS observed in SCS-BTA18-q animals. In summary, these results indicated *RELB *as an interesting positional and functional candidate gene for the SCS-BTA18-QTL, but further studies are needed to investigate the role of *RELB *within the SCS-BTA18-QTL and to confirm the results. A survey of polymorphisms within the *RELB *locus would help to clarify if *RELB *itself or other genes regulating *RELB *are causal for the SCS-BTA18-QTL.

## Conclusions

Primary bovine mammary gland epithelial cells sampled from marker selected half-sib heifers inheriting alternative paternal QTL alleles of a confirmed QTL for SCS showed distinct responses to pathogen challenge with heat-inactivated *E. coli *and *S. aureus *during time-course. The individual immune response of both, SCS-BTA18-Q and SCS-BTA18-q cells indicates that the established *in vitro *test system can reflect genetically determined differences in molecular mechanisms affected by the SCS-BTA18-QTL in response to pathogen challenge and that the underlying mechanisms of the SCS-BTA18-QTL might be attributed to immune functions. The early and linear up-regulation of cytokines in SCS-BTA18-Q cells suggests a superior immune response in SCS-BTA18-Q cells compared to SCS-BTA18-q cells. Especially the up-regulation of *RELB *and other genes involved in the non-canonical NF-kappaB signaling in SCS_BTA18-Q cells highlighted *RELB *as a positional and functional candidate gene affected by the SCS-BTA18-QTL. Future analyses of lymph node and parenchyma samples obtained from the genetically divergent half-sib heifers and a comparison to conventionally selected heifers will allow to more accurately define the molecular mechanisms specifically affected by the SCS-BTA18-QTL and could provide new insights into molecular mechanisms commonly involved in the response to pathogens in mammary gland epithelial cells.

## Competing interests

The authors declare that they have no competing interests.

## Authors' contributions

BB performed the microarray and bioinformatic analyses and drafted the manuscript. BGZ, OW and HHDM designed and coordinated the cell culture experiments and performed the Real-Time PCR analyses. CK and MS devised the design of the study, coordinated the study and participated in the interpretation of the data and critically revised the manuscript. SP, AH and DR participated in the microarray analyses and AH performed the miroarray experiments. All authors read and approved the final manuscript.

## Supplementary Material

Additional file 1 **Differentially expressed between time points 1 h and 24 h in un-inoculated control cells**. Lists of differentially expressed genes between time points 1 h and 24 h of un-inoculated control cells. Gene symbol, log_2 _fold changes as well as Entrez gene names are provided for each of the two paternally inherited SCS-BTA18-QTL alleles. Genes were selected on the basis of FDR adjusted p-values of q ≤ 0.05. Click here for file

Additional file 2 **List of genes showing a significant co-expression in time-course after inoculation with heat inactivated *E. coli *and *S. aureus *in SCS-BTA18-Q and SCS-BTA18-q cells, respectively**. Significantly co-expressed genes in *S. aureus *and *E. coli *inoculated SCS-BTA18-Q and SCS-BTA18-q cells identified using the clustering algorithm implemented in the short time-series expression miner STEM [28,29] (version 1.3.6). Only genes with a fold change of log_2 _fc ≥ 0.75 in time-course were considered. Significance was assessed based on the non-random co-expression of genes by comparing the number of genes assigned to a specific co-expression profile model to the expected number of genes assigned to the co-expression profile model quantified by permutation. The number of the profile, the human gene symbol and the log fold changes for time points 0 h, 1 h, 6 h and 24 h are shown. Click here for file
